# The *IL-6* rs1800795 and rs1800796 polymorphisms are associated with coronary artery disease risk

**DOI:** 10.1111/jcmm.15246

**Published:** 2020-05-06

**Authors:** Shuai Lu, Ya Wang, Yijun Wang, Jing Hu, Wu Di, Shuangye Liu, Xiaohui Zeng, Guo Yu, Yan Wang, Zhaohui Wang

**Affiliations:** ^1^ Department of Cardiology Union Hospital Tongji Medical College Huazhong University of Science and Technology Wuhan China; ^2^ School of Basic Medical Science Zhengzhou University Zhengzhou China; ^3^ School of Mathematical Science Tongji University Shanghai China

**Keywords:** coronary artery disease, IL6 rs1800795, IL6 rs1800796, polymorphism

## Abstract

Studies examining the associations between the *interleukin‐6* (*IL‐6*) rs1800795 and rs1800796 gene polymorphisms and risk of coronary artery disease (CAD) remain controversial. Our aim was to evaluate the accurately determine role of these two polymorphisms in CAD risk. PubMed, Embase, VIP, Wan fang and China National Knowledge Infrastructure databases were searched. The odds ratios (ORs) and 95% confidence intervals (CIs) were calculated. The trial sequential analysis (TSA) was conducted, and bioinformatics tools were employed. A total of thirty‐seven articles were obtained. For the IL‐6 rs1800795 polymorphism, 9411 CAD patients and 3161 controls were included, 4720 patients with CAD, and 5000 controls were included for the IL‐6 rs1800796 polymorphism. In the pooled analysis, significant associations were only observed for the rs1800796 polymorphism (allelic: OR [95%CI] = 1.28 [1.13, 1.44], dominant: OR [95%CI] = 1.35 [1.17, 1.57], recessive: OR [95%CI] = 1.35 [1.18, 1.55], heterozygote: OR [95%CI] = 1.26 [1.15, 1.37], homozygote: OR [95%CI] = 1.62 [1.23, 2.13]). Significant associations were detected in the Asian and Mongoloid populations and ‘more than 500’ subgroup for the rs1800795 polymorphism. TSA confirmed the true‐positive results for the rs1800796 polymorphism. The bioinformatics analysis showed that the two polymorphisms played important roles in the gene transcription. The *IL‐6* rs1800796 polymorphism is associated with an increased susceptibility to CAD and is a risk factor for CAD. The *IL‐6* rs1800795 polymorphism is associated with an increased risk of CAD in Asians, particularly in Chinese, and a decreased risk of CAD in an African population is remarkably observed.

## 1 INTRODUCTION

Coronary artery disease (CAD) is the leading cause of death both in developed and developing countries.^1^, ^2^ The aetiology of CAD remains obscure. Environmental and genetic factors, as well as the interactions between them, play a crucial role in the pathophysiology of CAD.^3^, ^4^ The heritability of CAD was estimated to range from 40%‐60% based on family and twin studies.^5^ Furthermore, the greatest genetic influence was observed on early‐onset CAD events,^6^ which implies a more vital role for genetic factors in determining CAD risk. Genotyping common single nucleotide polymorphisms (SNPs) within a potential CAD–related gene is an essential and efficient method to detect genetic risk markers, and many significant SNPs associated with CAD risk have been reported, such as *matrix metalloproteinase‐9*,^7^ *interleukin‐27* ^8^ and *Toll‐like receptor 4*.^9^

Inflammation plays a key role in the pathophysiology of CAD by promoting the development of atherosclerosis.^10^ As a pro‐inflammatory and immune‐regulatory cytokine, IL‐6 plays an important role in the genesis and maintenance of the inflammatory response in atherosclerosis. The *IL‐6* gene is located on chromosome 7p21‐24 and comprises 5 introns and 6 exons.^11^ Many SNPs in the *IL‐6* gene related to CAD risk have been reported, including *IL‐6*‐174G/C,^12^ *IL‐6*‐572C/G,^13^ *IL‐6*‐597G/A,^14^ *IL‐6*‐634C/G ^15^ and *IL‐6*+2954G/C ^16^; however, some of them were not associated with CAD risk (IL6‐597G/A and +2954G/C) or only one study reported the increased risk of CAD (IL‐6‐634C/G). Among them, two common polymorphisms (*IL‐6* rs1800795 −174G/C and *IL‐6* rs1800796 −572C/G) have been extensively investigated; however, the results were inconclusive. Several previous studies have been conducted in an attempt to draw significant conclusions, but the limitations in sample size and potential false‐positive results caused by systematic errors may bias the results. We therefore performed a study to more accurately determine associations between *IL‐6* polymorphisms and CAD risk; in addition, the bioinformatics analysis was conducted to explore the potential molecular mechanism.

## 2 METHODS

### 2.1 Identification of the related studies

A comprehensive document retrieval procedure was conducted to identify for all relevant studies published prior to October 2019. PubMed, Embase, VIP, Wan fang and China National Knowledge Infrastructure databases were thoroughly searched by the first three investigators to identify potential studies examining the associations between polymorphisms in the *interleukin‐6 (IL‐6)* gene and coronary artery disease. The terms ‘coronary artery disease’, ‘coronary heart disease’, ‘myocardial infarction’, ‘CAD’, ‘heart disease’, ‘*interleukin‐6’, ‘IL‐6*’, ‘polymorphism’, ‘variant’ and ‘polymorphisms’ were used. The citations of review articles and all eligible studies were also browsed for additional potentially relevant study data. In addition, the language of the published studies was restricted to English.

### 2.2 Inclusion and exclusion criteria

For inclusion in our analysis, studies must have met the following inclusion criteria: (a) evaluation of the relationship between the *IL‐6* polymorphisms and coronary artery disease; (b) coronary artery disease was defined as 50% stenosis in the left main coronary artery, or multiple significant (≥70% stenosis) in more than one coronary artery^17^; (c) a case‐control or cohort design; (d) genotype distribution data were able to be acquired to calculate odds ratios (ORs) and 95% confidence intervals (CIs), particularly detailed data from the control group for testing Hardy‐Weinberg equilibrium. Exclusion criteria were as follows: (a) duplication of previous studies; (b) comments, reviews and editorials; (c) non‐English or non‐Chinese articles; and (d) studies lacking controls. Based on the inclusion and exclusion criteria, the first two authors independently reviewed the references and included the relevant studies. Any disagreement was solved by discussion with the third author (Wang).

### 2.3 Data extraction

For all included studies, the first two authors independently extracted the following data using a standardized form: first author's last name, year of publication, study country, study region, age and body mass index (BMI), source of the control population, genotyping method, sample size and genotype frequency of polymorphisms in the *IL6* gene in patients and controls. Disagreement was settled by rechecking the data or discussion with a third author.

### 2.4 Quality assessment

The quality of the included studies was independently assessed by all the authors according to a set of criteria that were modified based on the Newcastle‐Ottawa quality assessment scale (Table S1).

### 2.5 Statistical analysis

Hardy‐Weinberg equilibrium (HWE) was tested in control groups from each study using the chi‐squared test, and *P* < .05 was considered a significant departure from HWE. Odds ratios (ORs) and 95% confidence intervals (CIs) were calculated to evaluate the strength of the associations between *IL‐6* gene polymorphisms and coronary artery disease risk. Pooled ORs were calculated for the allelic model (*IL‐6* rs1800795: C versus G and *IL‐6* rs1800796: G versus C), recessive model (*IL‐6* rs1800795: CC versus CG + GG and *IL‐6* rs1800796: GG versus GC+CC), dominant model (*IL‐6* rs1800795: CC+CG versus GG and *IL‐6* rs1800796: GG+GC versus CC), heterozygote model (*IL‐6* rs1800795: CG versus GG and *IL‐6* rs1800796: GC versus CC) and homozygote model (*IL‐6* rs1800795: CC versus GG and *IL‐6* rs1800796: GG versus CC), respectively. Heterogeneity was evaluated using the Q statistic (significance level of *P* < .1) and I^2^ statistic (greater than 50% as evidence of a significant inconsistency). Heterogeneity between studies was evaluated with the I^2^ test, and a higher I^2^ values indicated higher levels of heterogeneity (I^2^ > 90%: extreme heterogeneity; I^2^ = 70% to 90%: substantial heterogeneity; I^2^ = 50% to 70%: moderate heterogeneity; I^2^ < 50%: low heterogeneity). In the heterogeneity evaluation, the fixed‐effects model was used when I^2^ < 50%, a random‐effects model was used if I^2^ = 50% to 90%, and the studies were not pooled if I2 > 90%. A sensitivity analysis was performed to detect heterogeneity by sequentially omitting each study. Additionally, analyses were performed in subgroups stratified by accordance with HWE, region, ethnicity, source of controls and sample size. The potential for publication bias was assessed with Begg's funnel plot and Egger's test. We applied the Bonferroni method,^18^ which controls for the false discovery rate (FDR), to adjust for multiple comparisons. All tests reported in this study were conducted with the REVMAN 5.3 software and the STATA software (version 12.0; State Corporation).

### 2.6 Trial sequential analysis

Systematic bias and random errors are inevitable when conducting a meta‐analysis because of the sparse data and repeated significance testing; moreover, trials with low methodological quality, publication bias and a small sample size may generate a false‐positive result. Trial sequential analysis (TSA) is an approach that provides the required amount of information (number of samples) and further reveals potentially false‐positive results in a meta‐analysis.^19^ Therefore, TSA was employed to calculate the required amount information for obtaining reliable data of our study.^20^, ^21^ The TSA was performed by anticipating a 10% relative risk reduction, an overall 5% risk of type I error and a statistical test power of 80%.

### 2.7 Bioinformatics analysis

Ensembl is a genome browser for vertebrate genomes that supports research in comparative genomics, evolution, sequence variation and transcriptional regulation, and this database provides the genomic context, genes and regulatory elements, flanking sequence, population genetics, phenotype data, sample genotypes, linkage disequilibrium and phylogenetic context of a single nucleotide polymorphism (http://asia.ensembl.org/index.html). SNPinfo is an important bioinformatics analysis tool that predicts SNP function. The SNPinfo database can help researches specify genes or linkage regions and select SNPs based on GWAS results, calculate linkage disequilibrium (LD) and predict functional characteristics of both coding and non‐coding SNPs (https://snpinfo.niehs.nih.gov/).^22^ In addition, the RNAfold web server is one of the core programmes of the Vienna RNA package that has been used to predict the minimum free energy of single sequences that influence the stability of the structure.^23^ Therefore, we conducted bioinformatics analyses using the aforementioned databases and methods to identify the potential molecular mechanisms for further research.

## 3 RESULT

### 3.1 Characteristics of the included studies

The PRISMA flow diagram of our analysis was shown in Table S2. Two hundred and thirty articles were retrieved by searching the international and Chinese databases. After removing duplicates and screening title and abstracts, 54 articles were subjected to the full‐text assessment and 12 articles were excluded due to the lack of detailed genotype distribution data. Finally, 37 articles^12^, ^13^, ^14^, ^16^, ^24^, ^25^, ^26^, ^27^, ^28^, ^29^, ^30^, ^31^, ^32^, ^33^, ^34^, ^35^, ^36^, ^37^, ^38^, ^39^, ^40^, ^41^, ^42^, ^43^, ^44^, ^45^, ^46^, ^47^, ^48^, ^49^, ^50^, ^51^, ^52^, ^53^, ^54^, ^55^, ^56^ were included in the qualitative and quantitative synthesis.

The characteristics of all included studies regarding the associations between *IL6* gene polymorphism and coronary artery disease are presented in Table 1. For the *IL‐6* rs1800795 polymorphism, 33 studies involving 9411 CAD patients and 3161 controls were included; 21 studies of 4720 patients with CAD and 5000 controls were included for the *IL‐6* rs1800796 polymorphism. Based on the modified Newcastle‐Ottawa Quality Assessment Scale, the score of each included study was greater than 7, which implied a sufficient methodological quality for analysis.

**TABLE 1:** Characteristics of included studies

Study	Year	Country	Region	Age		BMI		Control	Genotyping	Sample	Case			Control			Quality	HWE*
				CAD	Control	CAD	Control	Source	Method	Size	XX	XY	YY	XX	XY	YY	Score	
											GG	GC	CC	GG	GC	CC		
*IL6* rs1800795 polymorphism																		
Nauck^24^	2002	Germany	Europe	63.77 ± 9.89	58.30 ± 11.83	27.52 ± 4.04	27.44 ± 4.34	HB	PCR‐RFLP	3304	838	1238	499	230	355	144	8	.739
Georges^25^	2003	France	Europe	62 ± 10	61 ± 7	26.8 ± 3.6	26.6 ± 5.0	PB	PCR‐SSCP	495	124	223	82	25	25	16	9	.064
Yang^26^	2004	China	Asian	55 ± 14	52 ± 18	26.0 ± 3.3	23.1 ± 2.8	HB	PCR‐RFLP	295	110	2	0	179	4	0	8	.881
Sekuri^28^	2007	Turkey	Asian	46.3 ± 7.8	44.3 ± 7.2	26.5 ± 2.8	24.3 ± 2.6	PB	PCR‐RFLP	220	61	49	5	57	41	7	8	.919
Sarecka^30^	2008	Poland	Europe	43.0 ± 5.5	42.3 ± 6.5	26.7 ± 4.4	25.4 ± 3.5	PB	PCR‐RFLP	263	35	74	33	36	64	21	8	.413
Banerjee^31^	2009	India	Asian	56.3 ± 12.1	56.0 ± 9.5	NA	NA	HB	PCR‐RFLP	442	159	43	8	171	57	4	9	.763
Rios1^47^	2010	Brazil	South America	55.7 ± 7.9	51.8 ± 8.4	NA	NA	HB	PCR‐TaqMan	253	96	36	6	69	43	3	8	.217
Rios2^47^	2010	Brazil	South America	55.7 ± 6.7	53.0 ± 7.7	NA	NA	HB	PCR‐TaqMan	414	158	90	28	82	46	10	9	.323
Coker^48^	2011	Turkey	Asian	53.4 ± 9.5	53.9 ± 9.3	28.4 ± 3.7	28.1 ± 3.6	PB	PCR‐RFLP	402	102	56	9	141	81	13	9	.761
Ghazouani^33^	2011	Tunisia	Europe	58.1 ± 12.0	56.7 ± 14.12	27.08 ± 4.20	25.22 ± 2.35	HB	PCR‐RFLP	824	298	110	10	297	102	7	9	.602
Vakili^49^	2011	Iran	Asian	NA	NA	NA	NA	PB	PCR‐TaqMan	900	153	234	63	202	229	19	9	.000
Fan^56^	2011	China	Asian	52.1 ± 6.8	52.3 ± 8.8	NA	NA	HB	PCR‐RFLP	214	84	0	0	129	1	0	8	.965
Liu^34^	2011	China	Asian	60.6 ± 12.7	61.3 ± 13.7	NA	NA	HB	PCR‐RFLP	276	123	3	0	148	2	0	8	.934
Bhanushali^35^	2013	India	Asian	48 ± 11	50 ± 11	NA	NA	HB	PCR‐SNaPshot^a^	250	77	20	3	121	25	4	8	.068
Phulukdaree1^36^	2013	South Africa	Africa	NA	NA	NA	NA	HB	PCR‐RFLP	102	29	11	1	34	19	8	8	.062
Phulukdaree2^36^	2013	South Africa	Africa	NA	NA	NA	NA	HB	PCR‐RFLP	120	38	16	5	34	19	8	8	.062
Satti^38^	2013	Pakistan	Asian	46.4 ± 18.7	35.2 ± 17.4	25.9 ± 3.5	25.2 ± 3.5	PB	PCR‐RFLP	88	18	11	7	38	14	0	7	.262
Tong^50^	2013	China	Asian	61.4 ± 8.7	60.6 ± 9.6	23.2 ± 3.1	22.7 ± 2.8	HB	PCR‐TaqMan	667	201	87	38	220	98	23	9	.011
Zhang^37^	2013	China	Asian	NA	NA	NA	NA	HB	PCR‐HRM	506	221	10	0	264	11	0	9	.735
Elsaid^51^	2014	Egypt	Africa	53.54 ± 9.1	45.3 ± 7.2	NA	NA	PB	PCR‐TaqMan	208	26	55	23	0	49	55	8	.000
Galimudi^39^	2014	India	Asian	65 ± 5	64 ± 6	NA	NA	PB	PCR‐RFLP	400	72	102	26	113	69	18	9	.123
Hatzis1^40^	2014	Greece	Europe	NA	NA	NA	NA	HB	PCR‐RFLP	361	109	76	12	64	72	28	9	.733
Hatzis2^40^	2014	Greece	Europe	NA	NA	NA	NA	HB	PCR‐RFLP	285	36	71	43	67	57	11	8	.817
Sun^14^	2014	China	Asian	61.2 ± 8.5	56.4 ± 11.6	NA	NA	HB	PCR‐TaqMan	623	191	61	44	236	63	28	9	.000
Celik^16^	2015	Turkey	Asian	14.56 ± 1.73	13.91 ± 1.31	20.29 ± 3.59	19.78 ± 3.25	HB	PCR‐RFLP	82	24	12	0	29	16	1	7	.476
Li^41^	2015	China	Asian	NA	NA	NA	NA	HB	PCR‐RFLP	730	213	113	39	245	105	15	9	.382
Wang 42	2015	China	Asian	65.4 ± 8.4	64.9 ± 8.2	22.8 ± 2.9	22.6 ± 2.6	HB	PCR‐RFLP	804	153	171	78	176	187	39	9	.292
Yang^43^	2015	China	Asian	NA	NA	NA	NA	HB	PCR‐RFLP	820	198	163	49	239	146	25	9	.669
Hongmei^12^	2016	China	Asian	62.64 ± 8.43	61.43 ± 7.85	26.41 ± 2.56	25.75 ± 2.54	HB	PCR‐RFLP	571	256	19	0	282	14	0	8	.679
Mao^52^	2016	China	Asian	62.65 ± 9.72	56.82 ± 9.80	24.61 ± 4.16	21.57 ± 3.64	HB	PCR‐RFLP	584	142	45	37	267	63	30	7	.000
Jabir^53^	2017	Saudi Arabia	Asian	60.6 ± 8.85	47.7 ± 5.06	28.69 ± 4.34	30.89 ± 2.90	HB	PCR‐TaqMan	179	62	25	3	63	23	3	8	.620
Mitrokhin^54^	2017	Russian	Europe	70.37 ± 13.45	74.94 ± 7.43	30.71 ± 2.75	30.33 ± 6.09	HB	PCR‐TaqMan	314	62	100	36	32	58	26	9	.977
Chen^55^	2018	China	Asian	61.00 ± 10.49	60.37 ± 10.38	25.13 ± 8.12	23.47 ± 8.72	HB	Multiplex PCR	779	155	218	56	190	133	27	9	.581
*IL6* rs1800796 polymorphism										CC	CG	GG	CC	CG	GG			
Fu^45^	2006	China	Asian	61.8 ± 12.4	59.89 ± 14.35	NA	NA	HB	PCR‐RFLP	505	128	101	16	166	90	4	7	.034
Wei^27^	2006	China	Asian	61 ± 11	60 ± 10	NA	NA	HB	PCR‐RFLP	335	89	67	9	113	55	2	8	.095
Gao^29^	2008	China	Asian	65.2 ± 9.8	62.5 ± 11.8	NA	NA	HB	PCR‐RFLP	234	65	51	10	72	32	4	8	.850
Jia^46^	2010	China	Asian	NA	NA	NA	NA	HB	PCR	441	79	130	22	88	107	15	7	.021
Liang^32^	2010	China	Asian	57.6 ± 7.4	56.4 ± 8.2	26.4 ± 3.1	24.2 ± 2.6	HB	PCR‐RFLP	851	259	161	14	283	126	8	8	.156
Fan^56^	2011	China	Asian	52.1 ± 6.8	52.3 ± 8.8	NA	NA	HB	PCR‐RFLP	214	42	38	4	95	32	3	8	.875
Liu^34^	2011	China	Asian	60.6 ± 12.7	61.3 ± 13.7	NA	NA	HB	PCR‐RFLP	276	63	52	11	92	55	3	9	.107
Coker 48	2011	Turkey	Asian	53.4 ± 9.5	53.9 ± 9.3	28.4 ± 3.7	28.1 ± 3.6	PB	PCR‐RFLP	402	126	30	11	169	45	21	7	.000
Zhang^37^	2013	China	Asian	NA	NA	NA	NA	HB	PCR‐HRM	506	86	106	39	128	117	30	9	.675
Tong^50^	2013	China	Asian	61.4 ± 8.8	60.6 ± 9.7	23.2 ± 3.2	22.7 ± 2.9	HB	PCR‐TaqMan	667	179	110	37	180	120	41	7	.004
Sun^14^	2014	China	Asian	61.2 ± 8.5	56.4 ± 11.6	NA	NA	HB	PCR‐TaqMan	623	190	69	37	215	73	39	7	.000
Wang^42^	2015	China	Asian	65.4 ± 8.4	64.9 ± 8.2	22.8 ± 2.9	22.6 ± 2.6	HB	PCR‐RFLP	804	176	187	39	192	181	29	9	.119
Li^41^	2015	China	Asian	NA	NA	NA	NA	HB	PCR‐RFLP	729	132	165	68	166	155	43	9	.462
Fragoso^13^	2015	Mexico	South America	NA	NA	NA	NA	HB	PCR‐TaqMan	244	7	39	32	11	77	78	8	.163
Celik^16^	2015	Turkey	Asian	14.56 ± 1.73	13.91 ± 1.31	20.29 ± 3.59	19.78 ± 3.25	HB	PCR‐RFLP	82	25	10	1	42	3	1	7	.013
Mao^52^	2016	China	Asian	62.65 ± 9.72	56.82 ± 9.80	24.61 ± 4.16	21.57 ± 3.64	HB	PCR‐RFLP	584	97	110	17	147	176	37	8	.137
Hongmei^12^	2016	China	Asian	62.64 ± 8.43	61.43 ± 7.85	26.41 ± 2.56	25.75 ± 2.54	HB	PCR‐RFLP	572	87	134	55	135	129	32	8	.886
Chen^44^	2016	China	Asian	63.22 ± 9.40	53.81 ± 8.45	NA	NA	HB	PCR‐RFLP	399	72	98	27	108	83	11	8	.333
Jabir^53^	2017	Saudi Arabia	Asian	60.6 ± 8.85	47.7 ± 5.06	28.69 ± 4.34	30.89 ± 2.90	HB	PCR‐TaqMan	159	3	22	59	0	21	54	8	.159
Mitrokhin^54^	2017	Russian	Europe	70.37 ± 13.45	74.94 ± 7.43	30.71 ± 2.75	30.33 ± 6.09	HB	PCR‐TaqMan	314	0	16	182	2	10	104	7	.010
Chen^55^	2018	China	Asian	61.00 ± 10.49	60.37 ± 10.38	25.13 ± 8.12	23.47 ± 8.72	HB	Multiplex PCR	779	228	158	43	176	141	33	9	.539

Note

For rs1800795 polymorphism, XX, XY and YY represent GG, GC and CC, respectively; for rs1800796 polymorphism, XX, XY and YY represent CC, CG and GG, respectively.

Abbreviations: BMI, body mass index; CAD, coronary artery disease; HB, hospital based; NA, not available; PB, population based; PCR, polymorphism chain reaction‐restriction; PCR‐HRM, polymorphism chain reaction high‐resolution melting; PCR‐RFLP, polymorphism chain reaction‐restriction fragment length polymorphism; PCR‐SSCP, polymorphism chain reaction single‐strand conformation polymorphism; PCR‐TaqMan, polymorphism chain reaction‐restriction TaqMan polymorphism.

^a^ The polymorphism was determined by a variation of the allele termination assay reported by Bhanushali et al^35^

* *P* value for Hardy‐Weinberg equilibrium test in controls.

### 3.2 The pooled analysis of *IL‐6* polymorphisms and CAD risk

**FIGURE 1:**
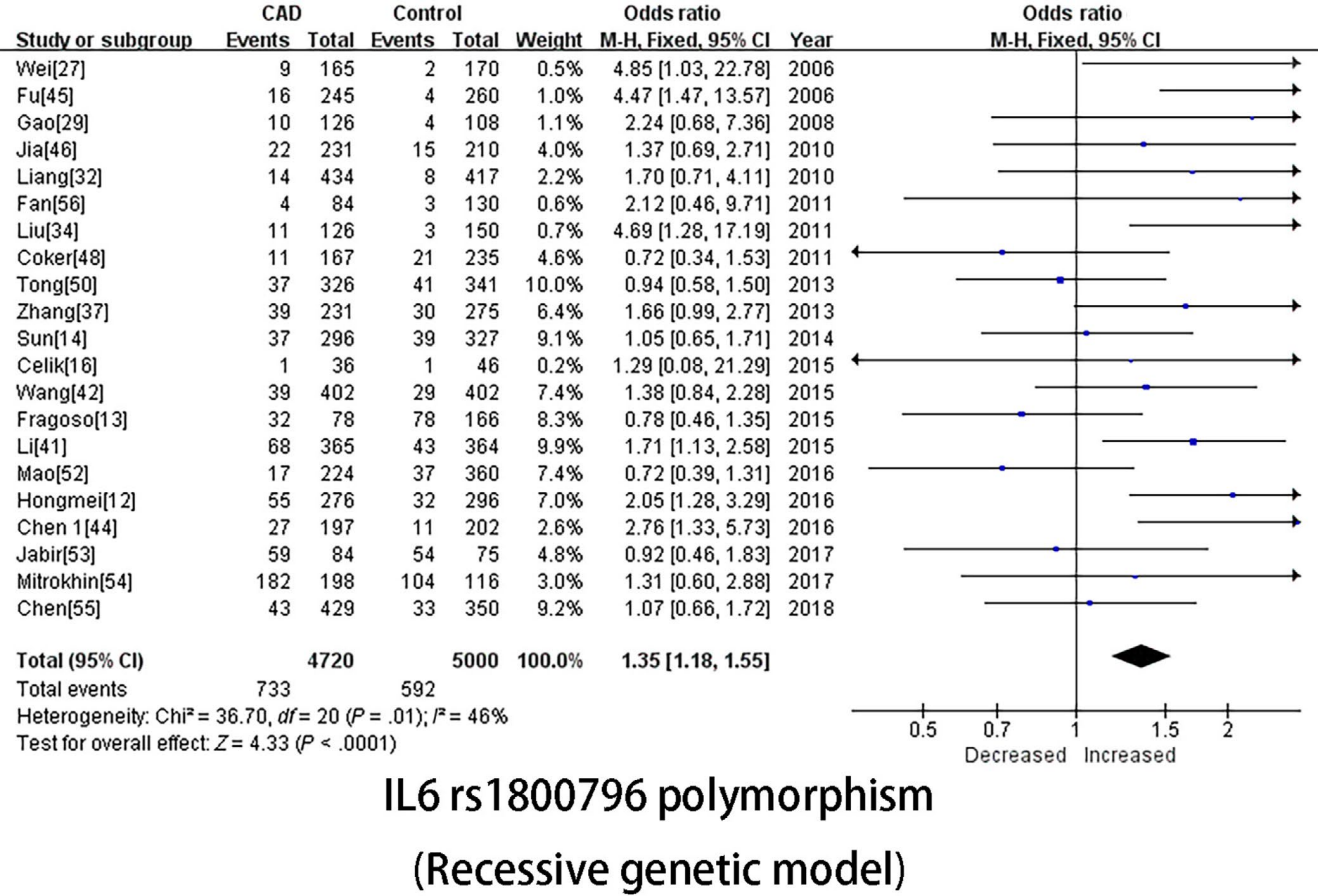
IL‐6 rs1800795 polymorphism (Recessive genetic model)

The main results of our analysis and the heterogeneity test of the associations between *IL‐6* gene polymorphisms and coronary artery disease risk are shown in Table 2. In the pooled analysis, no significant association was observed for the *IL‐6* rs1800795 polymorphism; significant associations with heterogeneity were detected in all five genetic models for the *IL‐6* rs1800796 polymorphism: allelic genetic model (OR [95% CI] = 1.28 [1.13, 1.44], *P* = 2^*^10^−4^), dominant genetic model (OR [95% CI] = 1.35 [1.17, 1.57], *P* = 2^*^10^−4^), heterogeneity genetic model (OR [95% CI] = 1.26 [1.15, 1.37], *P* = 2^*^10^−4^), recessive genetic model (OR [95% CI]=1.35 [1.18, 1.55], *P* = 2^*^10^−4^ (Figure 1)) and homozygote genetic model (OR [95% CI] = 1.62 [1.23, 2.13], *P* = .001).

**TABLE 2:** Pooled and Subgroup analysis of the associations between IL‐6 polymorphisms and CAD risk

Subgroup analysis	No. of the studies	Allelic genetic model			Dominant genetic model			Recessive genetic model			Heterozygote genetic model			Homozygote genetic model		
		OR [95%CI]	*P*_1_/Bon/FDR	*P*_2_/I2/EM	OR [95%CI]	*P*_1_/Bon/FDR	*P*_2_/I2/EM	OR [95%CI]	*P*_1_/Bon/FDR	*P*_2_/I2/EM	OR [95%CI]	*P*_1_/Bon/FDR	*P*_2_/I2/EM	OR [95%CI]	*P*_1_/Bon/FDR	*P*_2_/I2/EM
*IL6* rs1800795 polymorphism																
Pooled results	33	1.40 [1.12, 1.75]	.003/.015/.015	2*10^−4^/93%/R	1.21 [1.05, 1.40]	.01/.050/.0167	2*10^−4^/69%/R	1.34 [1.02, 1.76]	.04/.200/.040	2*10^−4^/78%/R	1.15 [1.01, 1.30]	.03/.150/.038	2*10^−4^/54%/R	1.48 [1.10, 2.00]	.01/.050/.017	2*10^−4^/78%/R
*Subgroup Results*																
HWE																
In accordance with HWE	28	1.31 [1.08, 1.59]	.007/.035/.035	2*10^−4^/88%/R	1.18 [1.00, 1.39]	.04/.200/.060	2*10^−4^/69%/R	1.30 [0.99, 1.72]	.06/.300/.060	2*10^−4^/69%/R	1.15 [0.99, 1.32]	.06/.300/.060	2*10^−4^/55%/R	1.40 [1.01, 1.94]	.04/.200/.060	2*10^−4^/75%/R
Departure from HWE	5	1.97 [1.01, 3.85]	.05/.25/.20	2*10^−4^/97%/R	1.34 [0.96, 1.88]	.09/.45/.20	.005/73%/R	1.49 [0.65, 3.40]	.35/1.00/.41	2*10^−4^/92%/R	1.14 [0.83, 1.58]	.41/1.00/.41	.03/63%/R	1.79 [0.86, 3.74]	.12/.60/.20	2*10^−4^/84%/R
Region																
Asian	21	1.84 [1.42, 2.39]	2*10^−4^/2*10^−4^/2*10^−4^	2*10^−4^/91%/R	**1.40 [1.24, 1.58]**	2*10^−4^/2*10^−4^/2*10^−4^	**.05/36%/R**	**1.99 [1.63, 2.42]**	2*10^−4^/2*10^−4^/2*10^−4^	**.20/22%/R**	**1.26 [1.11, 1.43]**	2*10^−4^/.002/2*10^−4^	**.10/30%/R**	**2.21 [1.78, 2.73]**	2*10^−4^/2*10^−4^/2*10^−4^	**.14/28%/R**
Europe	7	1.20 [0.87, 1.65]	.27/1/.800	2*10^−4^/90%/R	1.13 [0.80, 1.59]	.48/1/.800	2*10^−4^/82%/R	1.05 [0.65, 1.69]	.84/1/.840	2*10^−4^/81%/R	1.11 [0.85, 1.46]	.43/1/.800	.005/67%/R	1.15 [0.62, 2.11]	.66/1/.825	2*10^−4^/86%/R
Africa	3	**0.45 [0.26, 0.77]**	**.004/.02/.01**	**.06/64%/R**	0.33 [0.08, 1.33]	.12/.60/.15	.009/79%/R	**0.29 [0.17, 0.50]**	2*10^−4^/.00/.00	**.37/1%/R**	0.41 [0.11, 1.58]	.20/1.00/.20	.02/74%/R	0.11 [0.01, 1.37]	.09/.45/.15	0.009/79%/R
South America	2	0.97 [0.67, 1.41]	.88/1/.880	.17/46%/R	0.87 [0.53, 1.43]	.58/1/.725	.13/56%/R	1.50 [0.77, 2.91]	.23/1/.650	.84/0%/R	0.80 [0.48, 1.33]	.39/1/.650	.14/53%/R	1.45 [0.74, 2.85]	.28/1/.650	.99/0%/R
Ethnicity																
Caucasian	17	1.44 [1.10, 1.90]	.009/.045/.045	2*10^−4^/93%/R	1.20 [0.98, 1.47]	.07/.350/.150	2*10^−4^/73%/R	1.27 [0.89, 1.81]	.18/.900/.180	2*10^−4^/74%/R	1.15 [0.98, 1.37]	.09/.450/.150	.003/56%/R	1.40 [0.92, 2.14]	.12/.600/.150	2*10^−4^/79%/R
Mongoloid	12	1.97 [1.44, 2.70]	2*10^−4^/2*10^−4^/2*10^−4^	2*10^−4^/90%/R	**1.47 [1.31, 1.65]**	2*10^−4^/2*10^−4^/2*10^−4^	**.40/4%/R**	**2.07 [1.72, 2.50]**	2*10^−4^/2*10^−4^/2*10^−4^	**.94/0%/R**	**1.28 [1.11, 1.49]**	.001/.004/2*10^−4^	**.24/21%/R**	**2.28 [1.87, 2.77]**	2*10^−4^/2*10^−4^/2*10^−4^	**.92/0%/R**
African	4	0.52 [0.32, 0.86]	.01/.05/.050	.01/72%/R	0.49 [0.23, 1.05]	.07/.35/.125	.03/66%/R	0.46 [0.18, 1.17]	.10/.50/.125	.06/59%/R	0.55 [0.28, 1.11]	.10/.50/.125	.08/56%/R	0.24 [0.03, 1.61]	.14/.70/.140	.003/79%/R
Source of Controls																
Hospital based	25	1.48 [1.14, 1.94]	.004/.020/.020	2*10^−4^/94%/R	1.16 [0.99, 1.35]	.07/.350/.088	2*10^−4^/68%/R	**1.46 [1.10, 1.94]**	**.009/.045/.0225**	2*10^−4^ **/72%/R**	1.10 [0.96, 1.25]	.18/.900/.180	.004/48%/R	1.50 [1.08, 2.09]	.02/.100/.033	2*10^−4^/78%/R
Population based	8	1.18 [0.78, 1.80]	.43/1.0/.5375	2*10^−4^/91%/R	1.39 [0.98, 1.96]	.06/.3/.200	.001/72%/R	1.17 [0.57, 2.40]	.68/1.0/.6800	2*10^−4^/87%/R	1.34 [0.97, 1.84]	.08/.4/.200	.007/64%/R	1.37 [0.64, 2.92]	.41/1.0/.538	2*10^−4^/81%/R
Sample size																
Less than 300	14	1.21 [0.68, 2.18]	.52/1.0/.97	2*10^−4^/93%/R	1.05 [0.72, 1.54]	.80/1.0/.97	2*10^−4^/68%/R	1.02 [0.47, 2.21]	.97/1.0/.97	2*10^−4^/79%/R	1.04 [0.77, 1.41]	.80/1.0/.97	.03/46%/R	1.04 [0.77, 1.41]	.94/1.0/.97	2*10^−4^/78%/R
Between 300 and 500	7	1.11 [0.80, 1.53]	.54/1.0/.99	2*10^−4^/85%/R	1.05 [0.73, 1.51]	.80/1.0/.99	2*10^−4^/79%/R	0.92 [0.59, 1.43]	.71/1.0/.99	.02/61%/R	1.08 [0.76, 1.54]	.66/1.0/.99	.001/75%/R	1.08 [0.76, 1.54]	.99/1.0/.99	.001/73%/R
More than 500	12	**1.81 [1.33, 2.45]**	2*10^−4^/2*10^−4^/2*10^−4^	2*10^−4^/95%/R	**1.37 [1.17, 1.59]**	2*10^−4^/2*10^−4^/2*10^−4^	**.001/64%/R**	**1.95 [1.42, 2.67]**	2*10^−4^/2*10^−4^/2*10^−4^	2*10^−4^/78%/R	**1.22 [1.06, 1.40]**	.004/2*10^−4^/.004	**.03/48%/R**	**1.22 [1.06, 1.40]**	2*10^−4^/2*10^−4^/2*10^−4^	2*10^−4^/80%/R
*IL6* rs1800796 polymorphism																
Pooled results	21	**1.28 [1.13, 1.44]**	2*10^−4^/.001/2*10^−4^	2*10^−4^/68%/R	**1.35 [1.17, 1.57]**	2*10^−4^/.001/2*10^−4^	2*10^−4^/62%/R	**1.35 [1.18, 1.55]**	2*10^−4^/.001/2*10^−4^	**.01/46%/F**	**1.26 [1.15, 1.37]**	2*10^−4^/2*10^−4^/2*10^−4^	**.001/47%/F**	**1.62 [1.23, 2.13]**	**.001/.003/.001**	2*10^−4^/60%/R
*Subgroup results*																
HWE																
In accordance with HWE	14	**1.32 [1.15, 1.53]**	2*10^−4^/2*10^−4^/2*10^−4^	2*10^−4^/70%/R	**1.42 [1.19, 1.69]**	2*10^−4^/2*10^−4^/2*10^−4^	**.001/62%/R**	**1.48 [1.15, 1.91]**	.002/2*10^−4^/.0020	**.02/50%/R**	**1.33 [1.14, 1.55]**	2*10^−4^/2*10^−4^/.001	**.02/49%/R**	**1.78 [1.28, 2.47]**	.001/2*10^−4^/.001	**.002/60%/R**
Departure from HWE	7	1.18 [0.94, 1.47]	.15/.75/.283	.01/63%/R	1.22 [0.92, 1.62]	.16/.80/.283	.02/60%/R	1.16 [0.84, 1.61]	.37/1.00/.370	.21/29%/R	1.20 [0.92, 1.56]	.17/.85/.283	.07/48%/R	1.31 [0.82, 2.07]	.26/1.00/.325	.06/51%/R
Region																
Asian	19	**1.30 [1.15, 1.47]**	2*10^−4^/.001/2*10^−4^	2*10^−4^/69%/R	**1.36 [1.17, 1.58]**	2*10^−4^/.001/2*10^−4^	2*10^−4^/63%/R	**1.44 [1.16, 1.78]**	**.001/.005/.001**	**.02/45%/R**	**1.29 [1.13, 1.48]**	2*10^−4^/.001/2*10^−4^	**.008/50%/R**	**1.66 [1.26, 2.19]**	2*10^−4^/.002/2*10^−4^	2*10^−4^/61%/R
South America^a^	1	0.83 [0.55, 1.24]	.36/1.0/.6375	NA	0.72 [0.27, 1.93]	.51/1.0/.638	NA	0.78 [0.46, 1.35]	.38/1.0/.638	NA	0.80 [0.29, 2.21]	.66/1.0/.660	NA	0.64 [0.23, 1.81]	.4/1.0/.638	NA
Europe^a^	1	1.53 [0.73, 3.19]	.26/1.0/.325	NA	8.67 [0.41, 182.13]	.16/.8/.325	NA	1.31 [0.60, 2.88]	.5/1.0/0.5	NA	7.86 [0.34, 180.34]	.2/1.0/.325	NA	8.73 [0.42, 183.62]	.16/.8/.325	NA
Ethnicity																
Caucasian	16	**1.33 [1.17, 1.50]**	2*10^−4^/2*10^−4^/2*10^−4^	2*10^−4^/68%/R	**1.38 [1.19, 1.59]**	2*10^−4^/2*10^−4^/2*10^−4^	**.001/62%/R**	**1.54 [1.23, 1.93]**	2*10^−4^/2*10^−4^/2*10^−4^	**.02/47%/R**	**1.30 [1.14, 1.47]**	2*10^−4^/2*10^−4^/.0001	**.02/46%/R**	**1.78 [1.34, 2.36]**	2*10^−4^/2*10^−4^/2*10^−4^	2*10^−4^/62%/R
Mongoloid	5	1.02 [0.71, 1.48]	.90/1.0/.90	.08/52%/R	1.23 [0.50, 3.04]	.65/1.0/.813	.04/61%/R	0.88 [0.63, 1.23]	.47/1.0/.813	.82/0%/R	1.31 [0.52, 3.30]	.57/1.0/.813	.05/58%/R	0.73 [0.41, 1.31]	.30/1.0/.813	.41/0%/R
Source of controls																
Hospital based	20	**1.30 [1.16, 1.47]**	2*10^−4^/2*10^−4^/2*10^−4^	2*10^−4^/67%/R	**1.39 [1.19, 1.61]**	2*10^−4^/2*10^−4^/2*10^−4^	2*10^−4^/61%/R	**1.42 [1.16, 1.74]**	.001/2*10^−4^/.001	**.02/44%/R**	**1.31 [1.14, 1.50]**	2*10^−4^/2*10^−4^/.002	**.01/47%/R**	**1.70 [1.29, 2.24]**	2*10^−4^/2*10^−4^/2*10^−4^	**.001/58%/R**
Population based^a^	1	0.81 [0.56, 1.18]	.28/1.0/.5375	NA	0.83 [0.53, 1.31]	.43/1.0/.538	NA	0.72 [0.34, 1.53]	.39/1.0/.538	NA	0.89 [0.53, 1.50]	.67/1.0/.670	NA	0.70 [0.33, 1.51]	.37/1.0/.538	NA
Sample size																
Less than 300	6	1.46 [0.99, 2.14]	.05/.25/.083	.005/71%/R	1.46 [0.99, 2.14]	.01/.05/.050	.07/52%/R	1.37 [0.78, 2.40]	.28/1.00/.280	.12/42%/R	1.71 [1.06, 2.77]	.03/ .15/.075	.06/52%/R	1.75 [0.71, 4.31]	.22/1.00/.275	.08/50%/R
Between 300 and 500	5	1.35 [1.01, 1.80]	.04/.20/.067	.02/66%/R	1.35 [1.01, 1.80]	.04/.20/.067	.04/60%/R	1.56 [0.90, 2.69]	.11/.55/.110	.07/54%/R	1.40 [1.07, 1.83]	.01/.05/.050	.24/27%/R	2.17 [0.95, 4.93]	.06/.30/.075	.01/68%/R
More than 500	10	**1.21 [1.05, 1.39]**	**.008/.04/.010**	**.001/70%/R**	1.21 [1.05, 1.39]	.01/.05/.013	.006/61%/R	1.36 [1.07, 1.74]	.01/.05/.013	.03/50%/R	1.18 [1.03, 1.35]	.02/.10/.020	.10/38%/R	1.48 [1.09, 2.01]	.01/.05/.013	.002/65%/R

Note

Results with *P* < .05 even after the Bonferroni adjusted and a tolerable heterogeneity (I2 < 85%) were regarded as significant.

Abbreviations: BMI, body mass index; CI, confidence interval; EM, effect model; F, fixed effect model; OR, odds ratio; *P*1, *P* value for meta‐analysis; *P*2, *P* value for heterogeneity test; R, random effect model.

^a^ Only one study was included in the subgroup, and heterogeneity was not applicable.

Results with *P* < .05 even after the Bonferroni adjusted and a tolerable heterogeneity (I2 < 85%)

### 3.3 Subgroup analyses of the associations between *IL‐6* polymorphisms and CAD risk

Subgroup analyses were introduced to identify the source of heterogeneity and further reveal additional information about the associations between *IL‐6* polymorphisms and CAD risk. Table 2 summarizes the results of the subgroup analyses based on HWE, region, ethnicity, the source of controls and sample size.

For the subgroup in accordance with HWE, significant associations were only detected for the *IL‐6* rs1800796 polymorphism, and all five genetic models indicated strong associations with an increased OR compared with the pooled OR: allelic (OR [95% CI] = 1.32 [1.15, 1.53], *P* = 2^*^10^−4^), dominant (OR [95% CI] = 1.42 [1.19, 1.69], *P* = 2^*^10^−4^), recessive (OR [95% CI] = 1.48 [1.15, 1.91], *P* = 2^*^10^−4^), heterozygote (OR [95% CI] = 1.33 [1.14, 1.55], *P* = 2^*^10^−4^) and homozygote (OR [95% CI] = 1.78 [1.28, 2.47], *P* = .001) genetic models.

**FIGURE 2:**
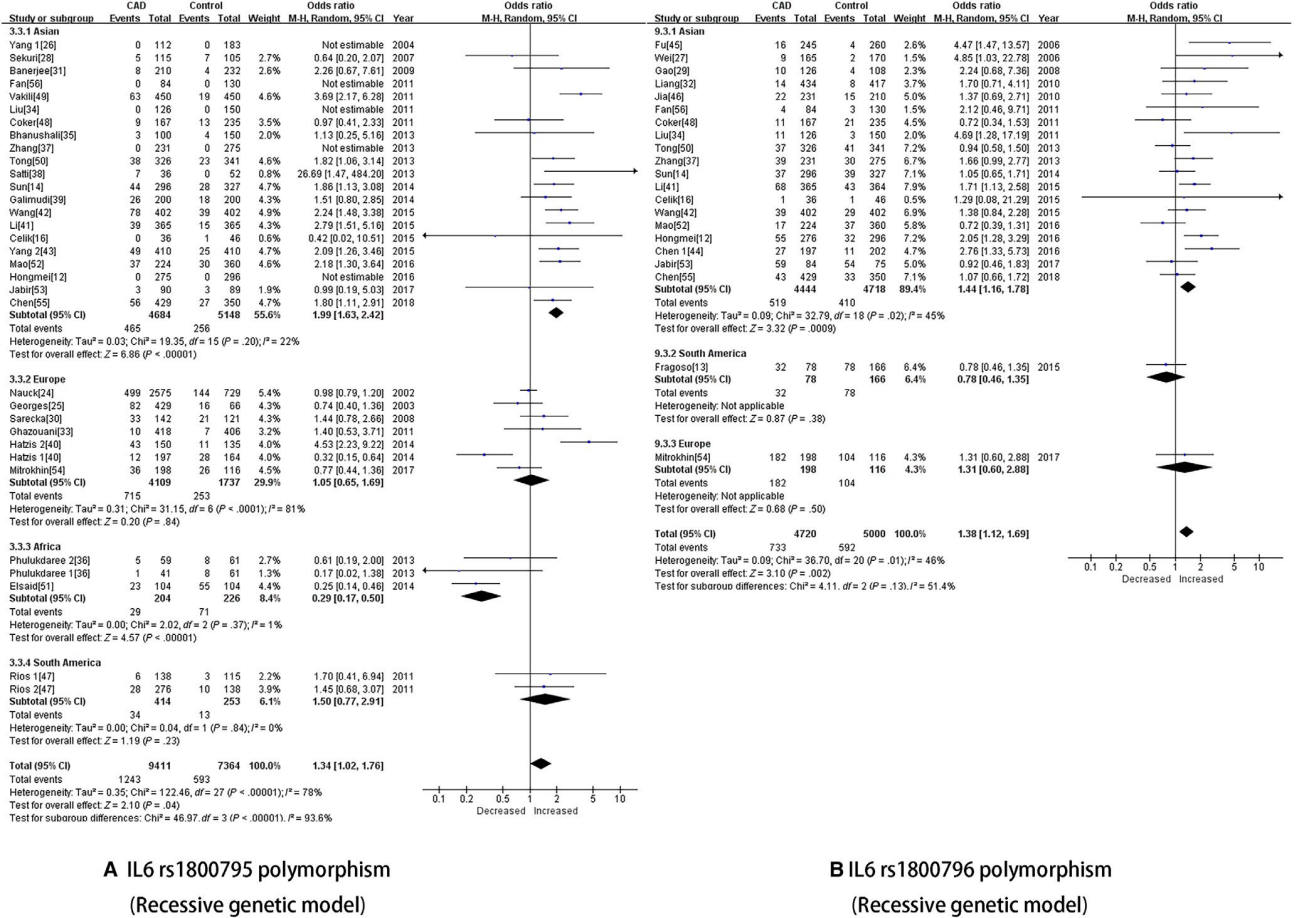
A, IL‐6 rs1800795 polymorphism (Recessive genetic model). B, IL‐6 rs1800796 polymorphism (Recessive genetic model)

In the analysis of the rs1800795 polymorphism stratified by region, significant associations with reduced heterogeneity in the Asian population were observed in the dominant (OR [95% CI] = 1.36 [1.17, 1.58], *P* = 2^*^10^−4^), recessive (OR [95% CI] = 1.44 [1.16, 1.78], *P* = .001) (Figure 2A), heterozygote (OR [95% CI] = 1.29 [1.13, 1.48], *P* = 2^*^10^−4^) and homozygote (OR [95% CI] = 1.66 [1.26, 2.19], *P* = 2^*^10^−4^) genetic models. In addition, decreased risks for the African population were remarkably identified in the allelic (OR [95% CI] = 0.45 [0.26, 0.77], *P* = .004) and recessive (OR [95% CI] = 0.29 [0.17, 0.50], *P* = 2^*^10^−4^) genetic models. Regarding the rs1800796 polymorphism, all five genetic models suggested a strong relationship with CAD risk in Asian volunteers (allelic: OR [95% CI] = 1.30 [1.15, 1.47], *P* = 2^*^10^−4^; dominant: OR [95% CI] = 1.36 [1.17, 1.58], *P* = 2^*^10^−4^; recessive: OR [95% CI] = 1.44 [1.16, 1.78], *P* = .001 (Figure 2B); heterozygote: OR [95% CI] = 1.29 [1.13, 1.48], *P* = 2^*^10^−4^; and homozygote: OR [95% CI] = 1.66 [1.26, 2.19], *P* = 2^*^10^−4^). Along with region as a geographic factor, ethnicity is also an important factor. In the Caucasian population, no association was observed for the rs1800795 polymorphism, but significant associations were widely observed with the rs1800796 polymorphism in all five genetic models (allelic: OR [95% CI] = 1.33 [1.17, 1.50], *P* = 2^*^10^−4^; dominant: OR [95% CI] = 1.38 [1.19, 1.59], *P* = 2^*^10^−4^; recessive: OR [95% CI] = 1.54 [1.23, 1.93], *P* = .001; heterozygote: OR [95% CI] = 1.30 [1.14, 1.47], *P* = 2^*^10^−4^; homozygote: OR [95% CI] = 1.78 [1.34, 2.36], *P* = 2^*^10^−4^). Regarding the Mongoloid population, significant associations were observed in the dominant (OR [95% CI] = 1.47 [1.31, 1.65], *P* = 2^*^10^−4^), recessive (OR [95% CI] = 2.07 [1.72, 2.50], *P* = 2^*^10^−4^), heterozygote (OR [95% CI] = 1.28 [1.11, 1.49], *P* = .001) and homozygote (OR [95% CI] = 2.28 [1.87, 2.77], *P* = 2^*^10^−4^) genetic models that implied a strong association between CAD risk and the *IL6* rs1800795 polymorphism.

In the subgroup analysis stratified by source of controls, significant associations were observed between CAD risk in the hospital‐based population and the rs1800795 polymorphism (recessive: OR [95% CI] = 1.46 [1.10, 1.94], *P* = .009) and the rs1800796 polymorphism (allelic: OR [95% CI] = 1.30 [1.16, 1.47], *P* = 2^*^10^−4^; dominant: OR [95% CI] = 1.39 [1.19, 1.61], *P* = 2^*^10^−4^; recessive: OR [95% CI] = 1.42 [1.16, 1.74], *P* = .001; heterozygote: OR [95% CI] = 1.31 [1.14, 1.50], *P* = 2^*^10^−4^; and homozygote: OR [95% CI] = 1.70 [1.29, 2.24], *P* = 2^*^10^−4^).

We stratified studies into three subgroups by sample size based on the modified quality scale score (less than 300, between 300 and 500, and greater than 500) to evaluate the effect of sample size on the associations between the two polymorphisms and CAD risk. In the greater than 500 subgroup, significant associations were observed between the rs1800795 polymorphism and CAD risk (dominant: OR [95% CI] = 1.37 [1.17, 1.59], *P* = 2^*^10^−4^; recessive: OR [95% CI] = 1.95 [1.42, 2.67], *P* = 2^*^10^−4^; heterozygote: OR [95% CI] = 1.22 [1.06, 1.40], *P* = .004; and homozygote: OR [95% CI] = 1.22 [1.06, 1.40], *P* = 2^*^10^−4^); for the rs1800796 polymorphism, a significant association was only observed in the allelic genetic model (OR [95% CI] = 1.21 [1.05, 1.39], *P* = .008). We discovered that a larger sample produced more significant associations with the two polymorphisms.

### 3.4 The sensitivity analysis of *IL‐6* polymorphisms and CAD risk

**FIGURE 3:**
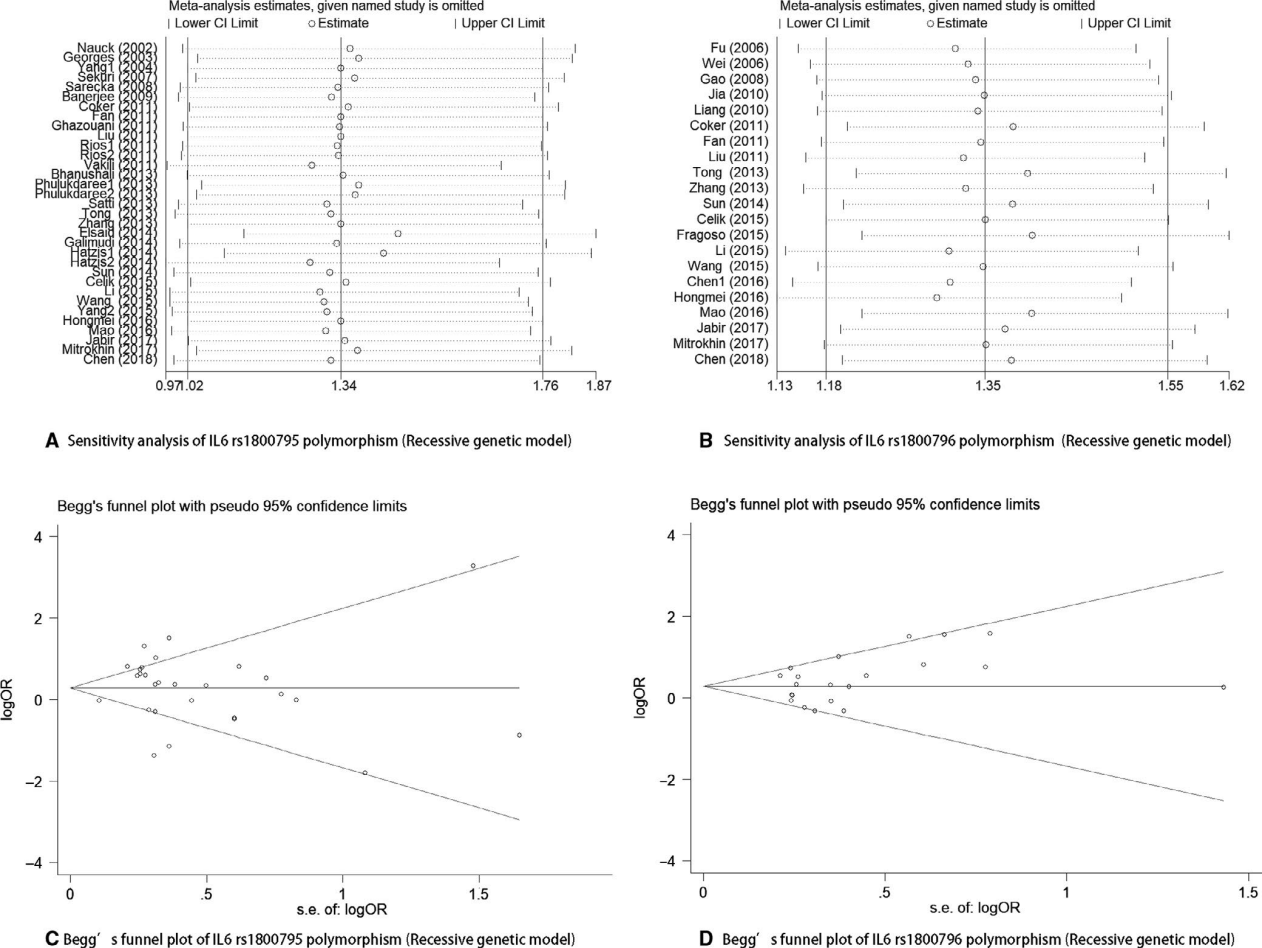
A, Sensitivity analysis of IL‐6 rs1800795 polymorphism (Recessive genetic model). B, Sensitivity analysis of IL‐6 rs1800796 polymorphism (Recessive genetic model). C, Begg's funnel plot of IL‐6 rs1800795 polymorphism (Recessive genetic model). D, Begg's funnel plot of IL‐6 rs1800796 polymorphism (Recessive genetic model)

A sensitivity analysis was conducted by sequentially omitting each individual study to detect the effect of each study on the results of the overall meta‐analysis. None of the studies changed the corresponding pooled ORs; thus, the results of our meta‐analysis were stable and reliable (Figure 3A‐B).

### 3.5 Publication bias

The *P* values for the Egger's test of the *IL‐6* rs1800795 and rs1800796 polymorphisms were .459 and .114, respectively; in addition, Begg's funnel plots of the two polymorphisms were symmetrical and all *P* values were greater than .05, indicating a lack of publication bias (Figure 3C‐D).

### 3.6 Trial sequential analysis

**FIGURE 4:**
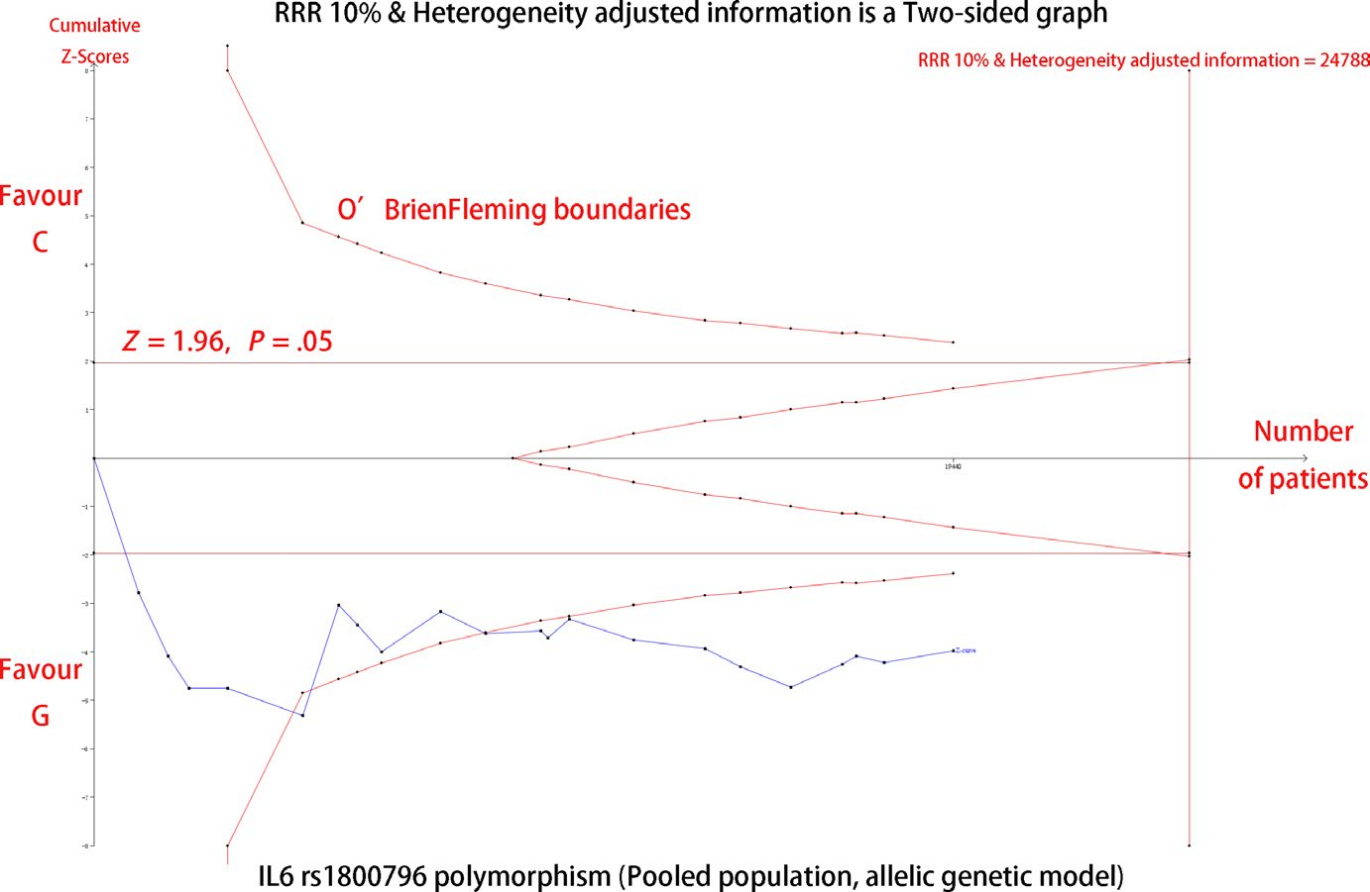
IL6 rs1800795 polymorphism (Pooled population, allelic genetic model)

A previous meta‐analysis of the associations between *IL‐6* polymorphisms and CAD risk reported negative results. For our pooled analysis of *IL‐6* rs1800795 and rs1800796 polymorphisms, significant associations were only observed for the *IL‐6* rs1800796 polymorphism. Hence, a trial sequential analysis was required to verify that our significant association was not a false‐positive result. Similar strength associations were discovered in five different genetic models. The allelic genetic model produced the best value and is a natural model of inheritance with a stronger genotype‐phenotype association, which also does not pre‐assume any interactions between the numbers of variant alleles. Therefore, we chose the allelic genetic model of the rs1800796 polymorphism to conduct the trial sequential analysis. The results of trial sequential analysis are shown in Figure 4. The x‐axis and y‐axis represent the number of patients and the cumulative Z score, respectively. Within the designed assumptions of confidence and effect size, the information size for the *IL‐6* rs1800796 polymorphism is 24 788, and the Z curves not only cross the statistical significance line (Z = 1.96, *P* = .05), but also cross the O’ Brien Fleming boundaries, indicating that the significance level of our study was a true‐positive result and the previously reported negative association was due to a lower number of volunteers.

### 3.7 Bioinformatics analysis

**FIGURE 5:**
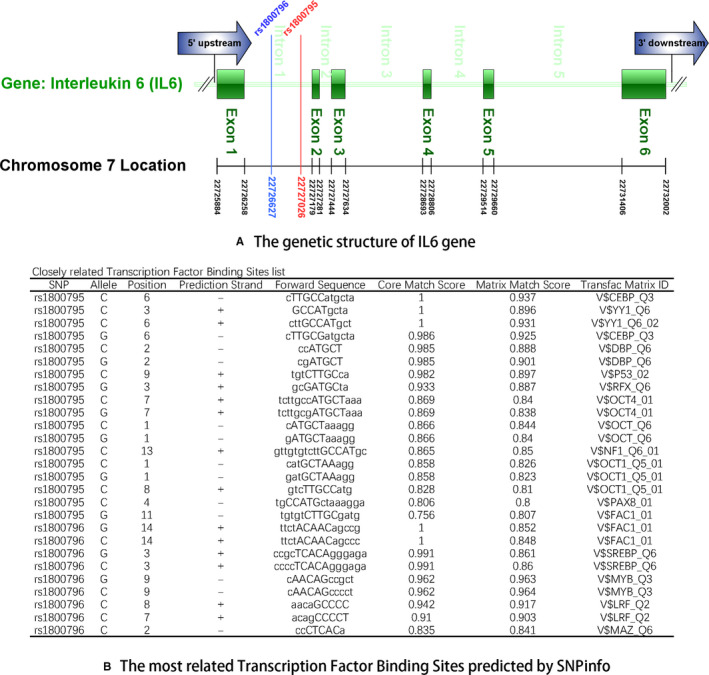
A, The genetic structure of IL‐6 gene. B, The most related Transcription Factor Binding Sites predicted by SNP ratio

**FIGURE 6:**
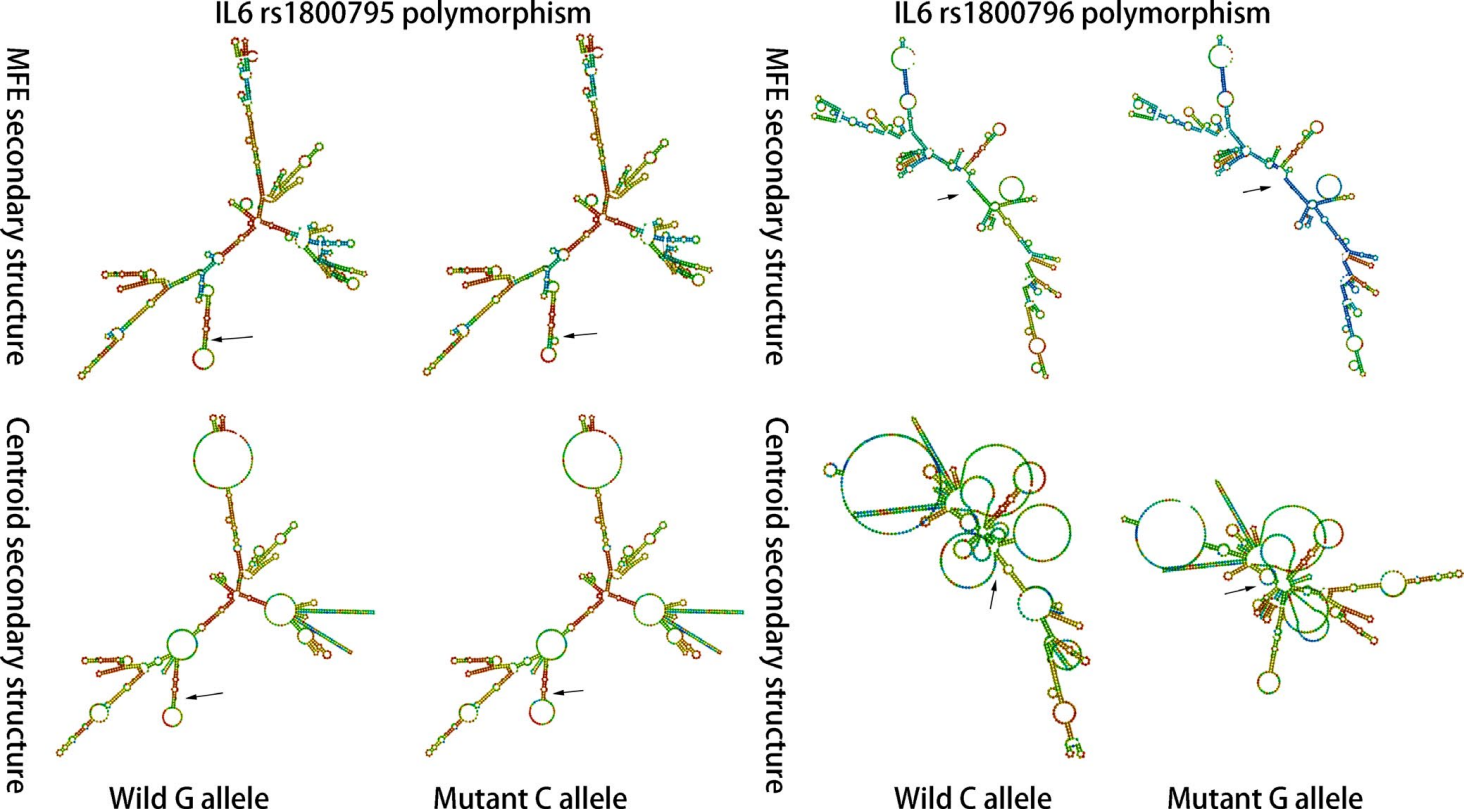
The RNAfold analysis of the IL‐6 polymorphisms

Based on the genomic context obtained from the Ensembl database, we constructed the summary genetic diagram for the rs1800795 and rs1800796 polymorphisms (Figure 5A). The two polymorphisms were both located in the promoter region near exon 2, implying that these sequences are potential transcription factor binding sites. Hence, we analysed the sequences of the two polymorphisms and the results from the SNPinfo database showed both polymorphisms are located in potential transcription factor binding sites (Figure 5B). In addition, the secondary structure of DNA at the rs1800795 and rs1800796 sequences was predicted using RNAfold. The minimum free energy (MFE) and the free energy of the thermodynamic ensemble (FETE) of the rs1800795 polymorphism were −142.50 kcal/mol and −170 kcal/mol for the wild G allele, and 141.60 kcal/mol and 169.58 kcal/mol for the mutant C allele, respectively. For the 1800796 polymorphism, the MFE and RFTE were −136.10 kcal/mol and −162.81 kcal/mol for the wild C allele, and −133.80 kcal/mol and −162.57 kcal/mol for the mutant G allele. Based on the predicted free energy of the two polymorphisms, the secondary structure of the two polymorphisms was determined. Compared with the wild allele, the mutant alleles of both polymorphisms caused a structure change, which were pointed with arrows in Figure 6.

## 4 DISCUSSION

In our study, two polymorphisms (rs1800795 and rs1800796) in the *IL‐6* gene were analysed for associations with CAD risk. The two common polymorphisms have been extensively studied in depth over the past few decades, providing sufficient enough data for a subgroup analysis designed to discover potential intriguing associations. Moreover, the two polymorphisms are located in the promoter region of the *IL‐6* gene, and may influence the expression of the *IL‐6* gene, and result in susceptibility to CAD. Several meta‐analyses have been conducted to explore the associations between the two polymorphisms and CAD risk, but the results were inconsistent. Significant associations between the *IL‐6* rs1800795 polymorphism and CAD risk were reported in some meta‐analyses,^57^, ^58^, ^59^ but not in the latest meta‐analysis reported by Liu et al^60^ Interestingly, the opposite result was obtained for the *IL‐6* rs1800796 polymorphism. The most recent studies by Song et al^61^ and Hou et al^57^ reported that this polymorphism may decrease the risk of CAD, which contradicts the conclusions of most previous meta‐analysis.^59^, ^60^, ^62^ We comprehensively reviewed these two studies and found that the opposite results may due to the relatively small sample size. Additionally, the adjusted alpha was not used to adjust for multiple tests, and thus, that conclusion that the result was a true positive is questionable. The controversial results from previous meta‐analysis and case‐control studies prompted us to examine the associations between the *IL6* rs1800795 and rs1800796 polymorphisms and CAD risk. Therefore, we chose these two common polymorphisms in the *IL6* gene to analyse the potential CAD risk.

No association between the *IL‐6* rs1800795 polymorphism and CAD risk with high heterogeneity was observed in the pooled results. Hence, we employed a detailed subgroup analysis to determine the potential sources of heterogeneity and associations. For the subgroup analysis stratified by region, significant associations with reduced heterogeneity were observed in the Asian population, which indicated an increased CAD risk for the rs1800795 polymorphism. Interesting results emerged in the analysis of the African subgroup and the decreased CAD risk for the mutant C allele and CC genotype were observed compared with the wild G allele and GC+CC genotypes, respectively. However, only three studies were included in the analysis of the African subgroup, and thus, we are sceptical about the conclusions and further studies are required. Similar findings were obtained for the Asian population. When stratified by ethnicity, the increased CAD risks for the rs1800795 polymorphism were observed in the Mongoloid population. The studies in the Mongoloid subgroup were performed in China, indicating that Chinese patients carrying the rs1800795 polymorphism would exhibit an increased risk of CAD. Moreover, high heterogeneity was significantly reduced when volunteers were stratified by region and ethnicity, indicating that these two factors are potential sources of high heterogeneity for the rs1800795 polymorphism. Sample size plays an important role in interpreting the conclusions of a case‐control study; thus, we conducted an analysis of subgroups stratified by sample size and discovered the increased CAD risk for the rs1800795 polymorphism in the larger sample size group. Therefore, if additional well‐designed studies are conducted, a lager sample size is required. The *IL‐6* rs1800796 polymorphism is associated with an increased risk of CAD. Extensive associations with an increased risk of CAD were observed in the pooled analysis and subgroup analyses. In addition, the trial sequential analysis confirmed the true‐positive results for the rs1800796 polymorphism, indicating that carriers of the rs1800796 mutant G allele are likely predisposed to CAD.

Coronary atherosclerosis is the main pathophysiological process in coronary artery disease, and inflammation plays a predominant role in atherosclerosis. As an important pro‐inflammatory cytokine, IL‐6 has been proven to be an independent risk factor for coronary artery disease and is expressed at relatively high levels in human atherosclerotic plaques.^63^, ^64^, ^65^ Considering the vital role of IL‐6 in atherosclerosis, the mechanism responsible for producing IL‐6 is a pivotal question. Researchers have not clearly determined whether polymorphisms in the *IL‐6* gene may also be an answer to this question. We conducted a bioinformatics analysis to predict the potential molecular mechanism. The two polymorphisms are located in the promoter region of the *IL‐6* gene, implying that the underlying mechanism occurs at the transcriptional level. The analysis of the SNPinfo database revealed potential transcription factor binding sites in the two polymorphisms, indicating the ability of these two polymorphisms to alter the expression of the *IL‐6* gene. In addition, an analysis of the sequence and secondary structure was performed using the RNAfold web server. The minimum free energy (MFE) and the free energy of the thermodynamic ensemble of the mutant alleles of the rs1800795 and rs1800796 polymorphisms were reduced compared with the wild alleles. The principle of minimum energy states that for a closed system, with constant external parameters and entropy, the internal energy will decrease and approach a minimum value at equilibrium.^66^ The mutant allele in a gene sequence may alter the free energy. The minimum free energy and the free energy of the thermodynamic ensemble are two thermodynamics parameters that have been used as a measure the required energy to reach the equilibrium for the stability of a sequence.^23^ The reduction implies that less energy is needed to form the secondary structure of the sequence containing the mutant allele, indicating that the sequence of the mutant alleles of the rs1800795 and rs1800796 polymorphisms is easier to disperse from the DNA double helix structure to serve as the template strand during transcription. Hence, these structural changes may affect the expression of the *IL‐6* gene. However, a bioinformatics prediction is not sufficient, and further fundamental research on the effect of the two polymorphisms on the transcription of the *IL6* gene is needed.

Several limitations existed in our study. First, only English and Chinese articles were included as a language restriction, which may bias the results. Second, the number of included studies was relatively small in some subgroups, such as the African population in the subgroup analysis of the *IL‐6* rs1800795 polymorphism, and thus, the results should be interpreted with caution. Third, only two common SNPs were evaluated in our study and other relevant SNPs in the *IL‐6* gene that are unknown or understudied may also have potential associations with CAD risk. Forth, the distance between the two common polymorphisms is relatively close, and the potential interactions between the two polymorphisms or other unknown polymorphisms need to be studied. In addition, the potential influence of environmental factors on genotype‐CAD associations is worth considering.

In conclusion, the *IL‐6* rs1800796 polymorphism is associated with an increased susceptibility to CAD and is a risk factor for CAD. In addition, the *IL‐6* rs1800795 polymorphism is associated with an increased risk of CAD in Asian, particularly in Chinese volunteers. Remarkably, a decreased risk of CAD was observed in the African population.

## CONFLICT OF INTEREST

The authors confirm that there are no conflicts of interest.

## AUTHOR CONTRIBUTION

Shuai Lu, Ya Wang and Zhaohui Wang wrote the main manuscript text. Yijun Wang and Shuai Lu prepared Figures 1 and 2. Wenhan Ma and Jing Hu prepared Figures 3 and 4. Shuangye Liu, Di Wu and Xiaohui Zeng draw the Figures 5 and 6. Yijun Wang made the Table 1. Shuai Lu, Ya Wang and Yijun Wang forged the Table 2. Guo Yu performed the statistical analysis of evaluating the strengths of associations between these two polymorphisms and CAD risk. Zhaohui Wang hosted the meeting addressing the disagreements. All authors reviewed and revised the manuscript.

## Supporting information

Table S1

Table S2

## Data Availability

All data included in this study are available upon request by contact with the corresponding author.
