# Supplementary material for: The IL-6 rs1800795 and rs1800796 polymorphisms are associated with coronary artery disease risk
Source: J Cell Mol Med. 2020 May 6;24(11):6191–207. doi: 10.1111/jcmm.15246 (PMC7294134; doi:10.1111/jcmm.15246)
Supplement: Supplementary file 1 — Table S1 [file JCMM-24-6191-s001.docx]

| **Table S1.** **Scale for methodological quality assessment.** | |
| --- | --- |
| Criteria | Score |
| 1.Representativeness of cases |  |
| Coronary artery disease diagnosed according to acknowledged criteria. | 2 |
| Mentioned the diagnosed criteria but not specifically described. | 1 |
| Not Mentioned. | 0 |
| 2.Source of controls |  |
| Population or community based | 3 |
| Hospital-based coronary artery disease free controls | 2 |
| Healthy volunteers without total description | 1 |
| Coronary artery disease free controls with related diseases | 0.5 |
| Not described | 0 |
| 3.Sample size |  |
| >500 | 2 |
| 300-500 | 1 |
| <300 | 0 |
| 4.Quality control of genotyping methods |  |
| Repetition of partial/total tested samples with a different method | 2 |
| Repetition of partial/total tested samples with the same method | 1 |
| Not described | 0 |
| 5.Hardy-Weinberg equilibrium (HWE) |  |
| Hardy-Weinberg equilibrium in control subjects | 1 |
| Hardy-Weinberg disequilibrium in control subjects | 0 |
