# Supplementary material for: The IL-6 rs1800795 and rs1800796 polymorphisms are associated with coronary artery disease risk
Source: J Cell Mol Med. 2020 May 6;24(11):6191–207. doi: 10.1111/jcmm.15246 (PMC7294134; doi:10.1111/jcmm.15246)
Supplement: Supplementary file 2 — Table S2 [file JCMM-24-6191-s002.docx]

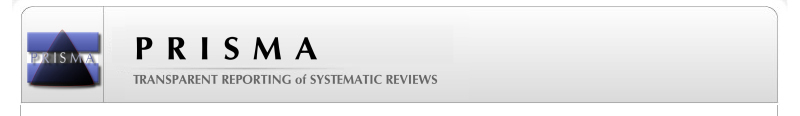
**Table S2. RISMA 2009 Flow Diagram**

## Included

Articles included in qualitative synthesis
(n = 36)

Full-text articles excluded,

Lack of detailed genotype distribution data
(n = 19)

Title and abstract excluded
(n = 61)

Title and abstract screened
(n = 66)

Full-text articles excluded,

Reviews, editorial

(n = 12)

Full-text articles assessed for eligibility
(n = 54)

Records identified through PubMed and Embase database searching
(n = 177)

Additional records identified through, VIP, Wangfang, and CNKI database searching
(n = 53)

Records after duplicates removed
(n = 127)

## Identification

## Eligibility

## Screening

Articles included in quantitative synthesis
(n = 37)

(33 studies for *IL6* rs1800795 polymorphism; 21 studies for *IL6* rs1800796 polymorphism)
